# Do Roads Reduce Painted Turtle (*Chrysemys picta*) Populations?

**DOI:** 10.1371/journal.pone.0098414

**Published:** 2014-05-23

**Authors:** Alexandra Dorland, Trina Rytwinski, Lenore Fahrig

**Affiliations:** Geomatics and Landscape Ecology Research Laboratory, Department of Biology, Carleton University, Ottawa, Ontario, Canada; The Australian National University, Australia

## Abstract

Road mortality is thought to be a leading cause of turtle population decline. However, empirical evidence of the direct negative effects of road mortality on turtle population abundance is lacking. The purpose of this study was to provide a strong test of the prediction that roads reduce turtle population abundance. While controlling for potentially confounding variables, we compared relative abundance of painted turtles (*Chrysemys picta*) in 20 ponds in Eastern Ontario, 10 as close as possible to high traffic roads (Road sites) and 10 as far as possible from any major roads (No Road sites). There was no significant effect of roads on painted turtle relative abundance. Furthermore, our data do not support other predictions of the road mortality hypothesis; we observed neither a higher relative frequency of males to females at Road sites than at No Road sites, nor a lower average body size of turtles at Road than at No Road sites. We speculate that, although roads can cause substantial adult mortality in turtles, other factors, such as release from predation on adults and/or nests close to roads counter the negative effect of road mortality in some populations. We suggest that road mitigation for painted turtles can be limited to locations where turtles are forced to migrate across high traffic roads due, for example, to destruction of local nesting habitat or seasonal drying of ponds. This conclusion should not be extrapolated to other species of turtles, where road mortality could have a larger population-level effect than on painted turtles.

## Introduction

Turtle populations have been declining over the past several decades [1]–[4]. Canada is home to eight species of freshwater turtles, each of which is listed as either endangered, threatened, or a species of special concern under the Canadian Species at Risk Act (SARA) in one or more of its regions of occurrence [5]. Turtle life history is characterized by high hatchling mortality, delayed sexual maturity, and high adult survivorship; thus, any threat that increases adult mortality has the potential to greatly impact the persistence of the population [6], [7].

There are several possible causes for turtle population declines. Turtle habitat destruction or alteration occurs through the construction or expansion of residential and commercial developments [2], [4], [8], [9]. Such development also increases the risk of predation on turtles and their nests due to associated increases in abundances of common predator species associated with humans, such as raccoons (*Procyon lotor*), foxes (*Vulpes vulpes*), coyotes (*Canis latrans*) and domestic dogs (*Canis lupus familiaris*), and cats (*Felis catus*) [10], [11]. Other turtle mortality factors include turtle bycatch in inland fisheries [12]–[14], mortality caused by recreational boating [7], [15], turtle collection for consumption purposes or for the pet trade [8], [16], and mortality on roads [8], [17]–[21]. Road mortality in particular is thought to be one of the primary causes of turtle population declines; for example, roads are reported as a principal threat to turtle populations in seven of the ten population status reports for turtle species listed on the SARA (Species at Risk Act) Public Registry [5].

Although roads are thought to have a negative effect on turtle abundance, there is very little direct evidence to date that roads actually cause declines in turtle populations. Rather, the inference that roads reduce turtle populations is based on indirect evidence, most notably altered sex ratios in turtle populations near roads. Male-biased sex ratios in turtle populations near roads [18]–[20], [22] have been interpreted as evidence that roads affect turtle population viability. Male-biased sex ratios are thought to be due to higher road mortality rates of female turtles than males because females often travel over land in search of suitable nesting sites, thus potentially coming into contact with roads [19], [23]. In addition, females commonly use the substrate along road edges for nesting sites, further increasing their chance of being killed by a vehicle [8], [24], [25]. Several studies have found proportionally more female turtles dead on roads than males (reviewed in Steen *et al*. [26]). It has been argued therefore that male-biased sex ratios are a sign of turtle populations in imminent danger of population decline [18], [22].

Despite the consensus that roads cause substantial adult mortality in turtles, as mentioned above, there is only one study empirically demonstrating that freshwater turtle population abundance or distribution is negatively affected by roads [27] (but see Boarman and Sazaki [28], Nafus *et al*. [29], and Crawford *et al*. [30] for evidence of negative road effects on desert tortoise (terrestrial) and diamondback terrapin (brackish water) populations). Fowle [27] found adult painted turtle population densities increased with distance from the highway. The strength of these results however are limited due to a small number of sampled ponds (n = 8), and a high correlation between pond area and distance from the highway, making it difficult to separate the road effect from the habitat amount effect on population density. At least two other studies have attempted to test this in painted turtles (*Chrysemys picta*) - Marchand and Litvaitis [18] and Steen and Gibbs [22] - but no significant road effects on population size were found. The authors offered two main explanations for this lack of effect. First, they suggested that perhaps the roads near the turtle populations were built too recently, thus not enough time had passed to produce an observable negative effect of road mortality on the population [9], [22]. Secondly, accurate abundance estimates for turtles are notoriously difficult to generate due to highly variable observability and catchability of turtles. The difficulty in estimating turtle populations sometimes results in confidence intervals around estimates that include zero, even when turtles have been observed [18], [22]. It also leads to small sample sizes in terms of the number of populations compared (e.g., near roads vs. far from roads); when more sampling effort is needed to estimate each population, fewer populations can be studied.

Our primary objective was to design a study that overcomes, to the extent possible, these difficulties and provides a strong test of the prediction that road mortality reduces painted turtle populations. We compared relative abundance of painted turtles in 20 ponds in Eastern Ontario, 10 as close as possible to high traffic roads (Road sites) and 10 as far as possible from high traffic roads (No Road sites). Only ponds and roads that had been in place for several decades were selected so that the effects of past road mortality on the populations would be observable [9]. We also measured variables that influence turtle detectability and we controlled for variance due to detectability in estimating turtle relative abundance. Our goal was to design a study with a high likelihood of detecting an effect of road mortality on painted turtle populations, if such an effect is present. If high traffic roads have a substantial impact on painted turtle populations, this should be evident in the current study. In addition to our main objective of comparing turtle relative abundance at Road and No Road sites, we compared the relative frequency of males and females captured at both site types. If females are killed more frequently on roads than males, we expected to see a higher relative abundance of males to females at the Road sites than at the No Road sites. Furthermore, we tested whether average body size and weight of captured turtles (particularly females) were lower at Road than at No Road sites. If adult females are killed by vehicles when they attempt to nest on road edges or to cross a road in search of a nesting site, then over time this mortality should lead to a decrease in turtle body size in the population, especially for females [29], [31].

## Methods

### Ethics Statement

This study was carried out in strict accordance with the guidelines from the Canadian Council on Animal Care (CCAC). The protocol for the full study was approved by the Carleton University Animal Care Committee (Protocol #: B10-32). *Chrysemys picta* is not a species at risk in Ontario [5]. Appropriate permits were obtained from the Ontario Ministry of Natural Resources (Authorization #: 1062791) which provided permission to conduct our research on *C. picta* at all study locations. No turtles were sacrificed for this research nor did they incur injury or death while in the traps or during handling.

### Site Selection

**Figure 1:**
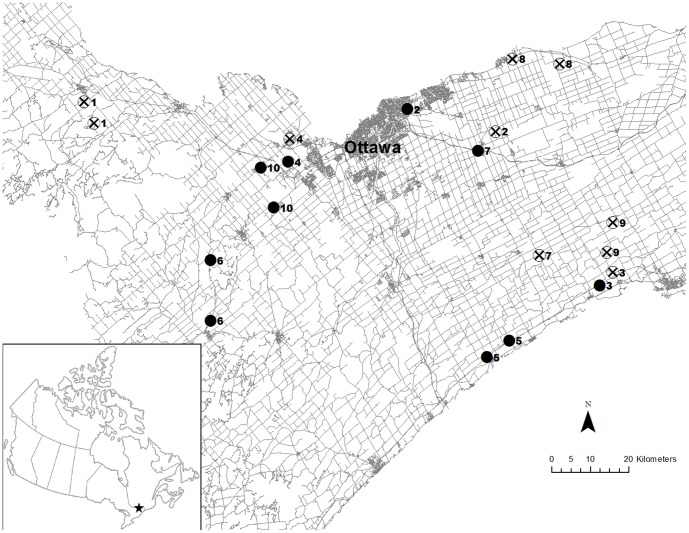
Sampled pond distribution across Eastern Ontario. Distribution of the 20 ponds (10 Road (solid black circles) and 10 No Road (crossed circles) sites) sampled across Eastern Ontario from 1 June to 28 August 2011. Ponds were paired for sampling based on geographical proximity. Paired sites share the same number (1–10) based on the order in which they were sampled.

We selected 20 permanent ponds in eastern Ontario, 10 of which were close to a freeway, highway or major arterial road (Road sites; mean distance to high traffic road  = 65 m±10 m (SE)), while the other 10 were as far as possible from any major roads (No Road sites; mean distance to closest major road  = 1517 m±285 m (SE)) (Figure 1). Eighteen of the sites were on privately owned land and permission was obtained from land owners to access the study locations (Table S1). Two of the study sites were on crown land in which no permission was needed to access ponds (Table S1). We used 300 m as the minimum distance from the pond to a major road for selection of No Road sites, as land based movements of most painted turtles occur within 300 m of the pond edge [10], [23], [32]–[34]. Three of the 10 No Road sites had no roads at all within a 300-m radius of the pond, three had one gravel road (one of these led to a dead end), and the remaining four No Road sites had one minor paved road within 300 m of the pond.

If road mortality due to collisions with vehicles reduces turtle populations, this negative effect should be most apparent at ponds near roads with high traffic volumes. Therefore, we selected ponds for the Road sites near major roads (i.e., arterial roads, highways, expressways, or freeways) with very high traffic volumes (6900 to 73932 AADT (Average Annual Daily Traffic); mean  = 20784 AADT). In contrast, AADT on the roads within 300 m of ponds in the No Road sites ranged from 50 to 500 AADT (mean  = 350 AADT) and included only minor roads (i.e., a local street or collector road, either paved or unpaved). Traffic volume was determined for seven of the 10 Road sites using 2008 data provided by the Ontario Ministry of Transportation [35] and for the three remaining Road sites using 2011 data provided by the City of Ottawa (unpublished data). Traffic data were not available for the minor roads within 300 m of ponds at the No Road sites; thus, we estimated AADT for these roads using the procedure described in Eigenbrod *et al*. [36]. We measured road density (m/km^2^) within a 300-m radius of each pond using the Ontario Road Network dataset [37].

We searched for ponds and highways that were well-established (i.e., neither the pond nor the highway had been recently created or built). The 20 ponds ranged in age from 25 to 90 years (mean  = 49 years), and the high traffic roadways associated with the Road sites ranged in age from 38 to 90 years (mean  = 55 years). Ages of ponds and roads were assessed using air photos from the Canadian National Air Photo Library and through personal communication with land owners.

Ponds ranged in size from 553 m^2^ to 19877 m^2^ (mean  = 6380 m^2^) and were a minimum of 4.5 km apart (Figure 1). The amount of forest in the surrounding landscape has been shown to affect turtle abundance in nearby wetlands [18], [38], [39], so we attempted to select sites such that the Road and No Road sites had similar amounts of forest within 300 m of the ponds (Road sites: 0.1 to 58.5%, mean  = 29.5% forest; No Road sites: 2.8 to 76.0%, mean  = 41.1% forest). We also attempted to keep the amount of crop cover within a 300-m radius similar between Road and No Road sites (Road sites: 0 to 35.8%, mean  = 17.1% crop cover; No Road sites: 0 to 59.8%, mean  = 25.2% crop cover). In addition, we selected only ponds that did not have another obvious water source, including wetlands, lakes, rivers, or other ponds, within 250 m (mean distance to nearest body of water  = 372 m±27 m (SE)). The amount of forest within a 300-m radius of each pond was determined using Ontario Ministry of Natural Resources thematic data (Forest cover: [40]) while the amount of crop cover was determined using aerial photos from 2008 and 2009 [41]). ArcMap 10.0 (ESRI, Redlands, California, USA) was used to analyse all geographic information systems data.

### *Chrysemys picta* Surveys

Painted turtle surveys took place between 1 June and 28 August 2011. For sampling purposes, ponds were paired based on geographical proximity with the exception of one pair that was separated by a relatively large distance compared to the other pairs (pair 7; Figure 1). Four of the ten pairs contained one Road and one No Road site while three pairs contained two Road sites and three pairs contained two No Road sites. Despite this, Road and No Road sites were sampled evenly throughout the sampling period.

Each pair of ponds was sampled twice a day for three consecutive days twice during the summer, for a total of six sampling days at each pond. Each sampling day was divided into four sampling periods, Morning 1 (M1, 08:00–10:20), Morning 2 (M2, 09:30–12:30), Afternoon 1 (A1, 12:30–14:30), and Afternoon 2 (A2, 13:15–16:45), such that each pond within a sampling pair was visited once in the morning and once in the afternoon each sampling day (Figure S1). The order of visits alternated each day so that the pond that was visited during M1 and A1 sampling periods on the first day was visited during the M2 and A2 sampling periods on the following day, and vice versa (Figure S1). After all 20 sites had been visited twice a day for three consecutive days between 1 June and 22 July 2011, the entire process was repeated, and each site was visited twice a day for another three consecutive days between 25 July and 28 August 2011. To ensure the same time interval for all sites between the first and second sampling periods, pairs of ponds were visited in the same order during the second sampling period as they were during the first, with the exception of two pairs that reversed order during the second sampling period.

Each sampling period began with a single, slow, meticulous, unidirectional search for turtles along the perimeter (<3 m from shore) of the pond either by canoe or by foot. We recorded all turtles seen, and we attempted to catch every turtle seen with a dipnet or by hand. On the first day of the 3-day period, directly following the perimeter search during the morning sampling period, two hoopnets (0.31 m diameter, 1.83×0.91 m wings, 3.81 cm square nylon netting) were installed at the pond. Each hoopnet was placed with the open end facing the shoreline and baited with a partially opened can of sardines [39], [42]. A floatation device was placed near the closed end of each net to allow trapped turtles access to the surface for air. The hoopnets were placed in areas where we had seen turtles during the perimeter search or, if no turtles had been seen, they were placed near areas that provided suitable basking habitat (including fallen logs or emergent rocks along the shoreline). Hoopnets were left in place for the duration of the three day period and were checked for turtles following the perimeter searches during each visit after initial set-up. The hoopnets were removed from the pond following the morning perimeter search on the last day of a three consecutive day period (Figure S1).

All turtles caught were weighed using a Starfrit 5 kg digital scale (±1 g), and their straight-line carapace length, width, and depth were measured using a transfer caliper (mm). Sex was determined using secondary sex characteristics: the male cloacal opening is located on the portion of the tail that extends past the posterior edge of the carapace while the female cloacal opening is located on the portion of the tail that does not extend past the posterior edge of the carapace. Additionally, males have much longer foreclaws than females [4]. Size at sexual maturity in painted turtles is highly variable [43]–[45]; therefore, we used the carapace length of the smallest identifiable male we sampled as the minimum size for assigning gender. That is to say, any non-male turtle that was larger than the minimum carapace length was classified as a female, and any turtle smaller than the minimum size was classified as a juvenile of unknown gender. Each captured turtle was given a unique carapace mark by drilling a small hole in the outer edge of two marginal scutes [46]. Turtles were released immediately after being weighed, measured, and marked. Any recaptures were recorded and released immediately.

### Local Site Characteristics

We carried out vegetation surveys from 25 July to 28 August 2011, once at each site. Marchand and Litvaitis [18] found that shoreline vegetation composition and percent surface cover by herbaceous-emergent vegetation were significantly related to turtle abundance. Therefore, we conducted a visual survey of the surface of each pond, recording the percent surface covered by open water, emergent vegetation, and submerged aquatic vegetation that reached, but did not break, the surface. We also conducted a visual survey of adjacent upland local habitat, including percent area covered by forest, shrubs, grass, and open ground within 5 m of the shoreline. We measured temperature, pH, and conductivity at random surface locations of each pond immediately following each turtle survey using a Hanna Instrument handheld tester (HI 98129). Pond depth was also measured using a weighted meter rope. We also measured pond visibility as the average visible depth (cm). If the water was perfectly clear and there was no vegetation obstructing the view, visibility was taken as the depth of the pond, to a maximum of 150 cm, as this was the maximum depth at which observations were made. Otherwise, visibility was measured at the centre of the pond by lowering a brick (similar in colour when wet to a turtle underwater) tied to a meter rope into the water.

## Data Analysis

**Figure 2:**
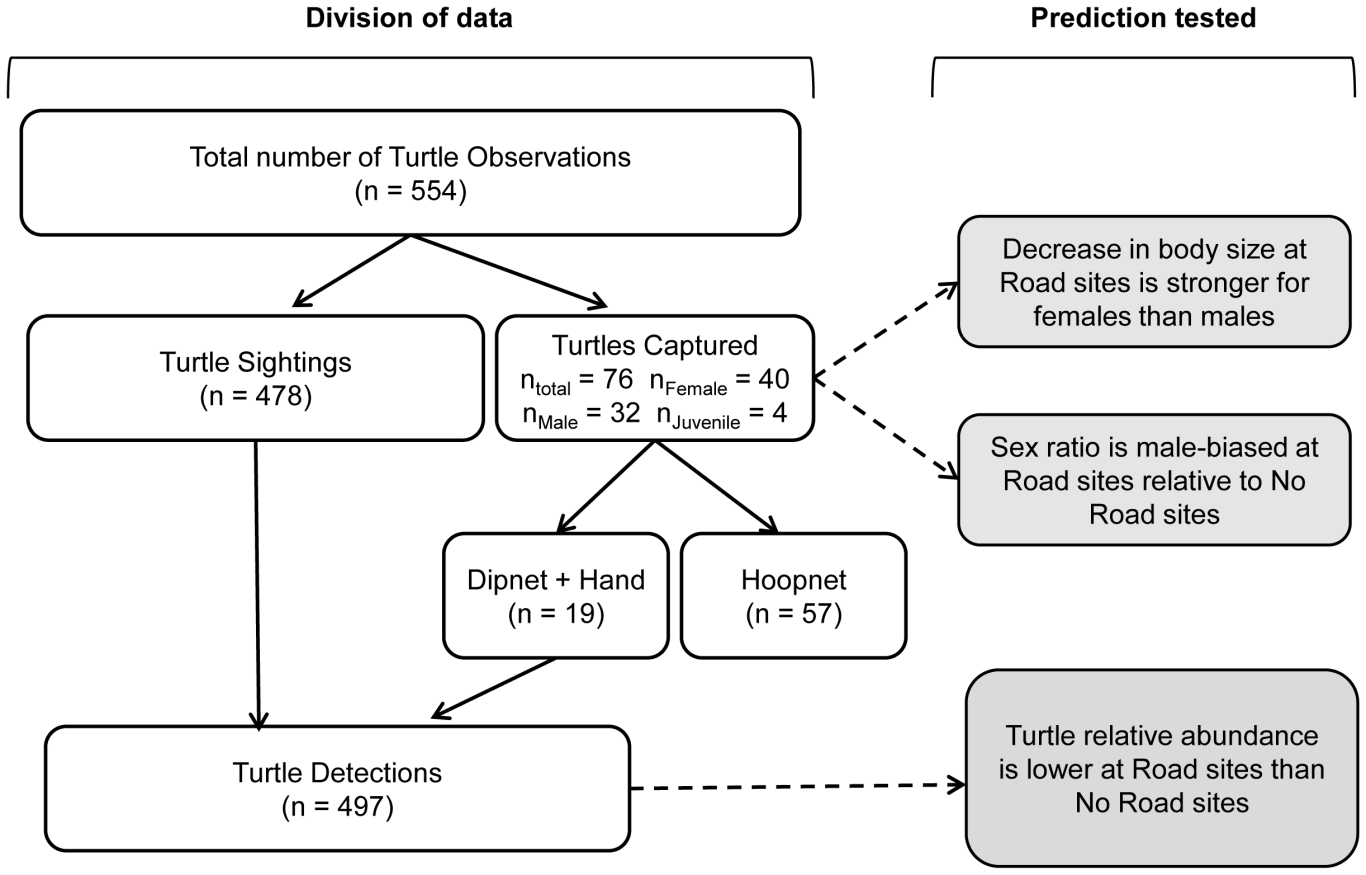
The division of data into groupings used for analyses. Data categories are shown in white boxes with solid black arrows showing which data are included in each of the categories. Predictions tested are shown in grey boxes with dashed arrows indicating the data category (in these cases the response variable) used in each.

Turtle observations were one of three mutually exclusive types: (1) Sightings, (2) Captures by dipnet or by hand, or (3) Captures by hoopnet. Data subsets were created using various combinations of these. “Turtle Detections” represented sightings and captures that were affected by detectability (see below), and included all turtle sightings and captures either by dipnet or by hand (i.e., types (1) + (2) above) (Figure 2). We used Turtle Detections as the response variable to test our main prediction that turtle relative abundance at Road sites is lower than at No Road sites. “Turtles Captured” included turtles that were captured either by dipnet, by hand, or by hoopnet, and individually marked (i.e., types (2) + (3) above) (Figure 2). We used Turtles Captured as the response variable to test the predictions that the sex ratio should be more male-biased at Road sites than at No Road sites and that a decrease in body size at Road sites (relative to No Road sites) should be stronger for females than for males.

### Potentially Confounding Variables

#### Local site and landscape characteristics

Although we tried to control for potentially confounding variables during site selection, there were still some variables potentially affecting turtle abundance that we were unable to completely control for. These included variables from two categories: landscape characteristics and local site characteristics. Potentially confounding landscape characteristics included: (1) percent area (within 300 m of each pond) covered by forest, (2) percent area (within 300 m of each pond) covered by crop, (3) percent area (within 300 m of each pond) covered by urban development, and (4) distance to nearest body of water. Potentially confounding local site characteristics included: (1) percent upland area (within 5-m radius of pond edge) covered by forest, (2) percent upland area (within 5-m radius of pond edge) covered by shrubs, (3) percent upland area (within 5-m radius of pond edge) covered by grass, (4) percent upland area (within 5-m radius of pond edge) covered by open ground, (5) percent surface water (at pond surface) covered by open water, (6) percent surface water (at pond surface) covered by emergent vegetation, and (7) percent surface water (at pond surface) covered by submerged aquatic vegetation. Preliminary analyses were conducted using a series of two-tailed t-tests to determine whether any of the potentially confounding variables differed significantly between the Road and No Road sites. We intended to include in further analyses any variable that differed significantly between the Road and No Road sites.

#### Turtle Detectability

Reliable estimates, even relative estimates, of freshwater turtle populations are difficult to obtain because of the many factors that can influence an observer's ability to detect turtles in the water. These include water clarity, water depth, and whether or not there is submerged vegetation obstructing the view to the bottom. These factors must be taken into account when estimating turtle populations. In addition, while we attempted to standardize the sizes of the ponds, there was inevitably variation in pond size which should also be controlled for when testing for a difference in turtle abundance between the Road and No Road sites. We thus created a single “Detectability” score as the product of pond size and visibility. Pond size was measured as the perimeter of the pond in meters. To validate the Detectability score, we conducted a simple linear regression of Turtle Detections on Detectability. To determine whether detectability was a potential confounding variable, we determined whether Detectability scores differed significantly between the Road and No Road sites. Finally, we included a Detectability co-variate in further analyses of Turtle Detections (the response variable) to control for variability due to detectability when testing for a difference in relative turtle abundance between Road and No Road sites.

### Effects of Roads on Relative Abundance, Sex Ratio, and Body Size

To test our main prediction that turtle relative abundance at Road sites is lower than at No Road sites, we conducted a multiple regression using Turtle Detections as the response variable and Detectability and Site Type (Road vs. No Road) as predictors. In addition to the Site Type predictor, we investigated other potential road-related predictors by running two additional multiple regressions using Turtle Detections as the response variable and Detectability and (1) road density (m/km^2^) or (2) total traffic volume (AADT), both measured within 300 m of the pond. Total traffic volume was calculated as the length of the road within the 300-m radius area multiplied by its traffic volume [35]. To test the predictions that the sex ratio was male-biased at Road sites relative to No Road sites, we performed a chi-square test of independence on the number of males and females captured (Turtles Captured with juveniles removed) at Road and No Road sites. Finally, to test our prediction that the decrease in body size at Road sites was stronger for females than males, we conducted four one-tailed t-tests using Turtles Captured, with juveniles removed, as the response variable: two t-tests comparing average weight (in g) of female and male turtles, respectively, at Road and No Road sites, and two t-tests comparing average straight-line carapace length (in mm) of females and males, respectively, at Road and No Road sites.

All statistical analyses were conducted using SPSS version 19.0. All data and residuals were screened for normality and transformed as necessary. The response variable Turtle Detections was log transformed (log[Turtle Detections +1]) for all analyses. Before taking the log of Turtle Detections we had to add a constant (here, 1) to all values, as we could not take logs of zero values.

## Results

Turtle observations totalled 554 (246 at Road sites and 308 at No Road sites); 478 were sightings, 57 were caught by hoopnet, and only 19 were caught by dipnet or by hand as sighted turtles were extremely difficult to capture (Figure 2). Of the turtles that were caught, 40 were female, 32 were male, and 4 were juvenile.

**Figure 3:**
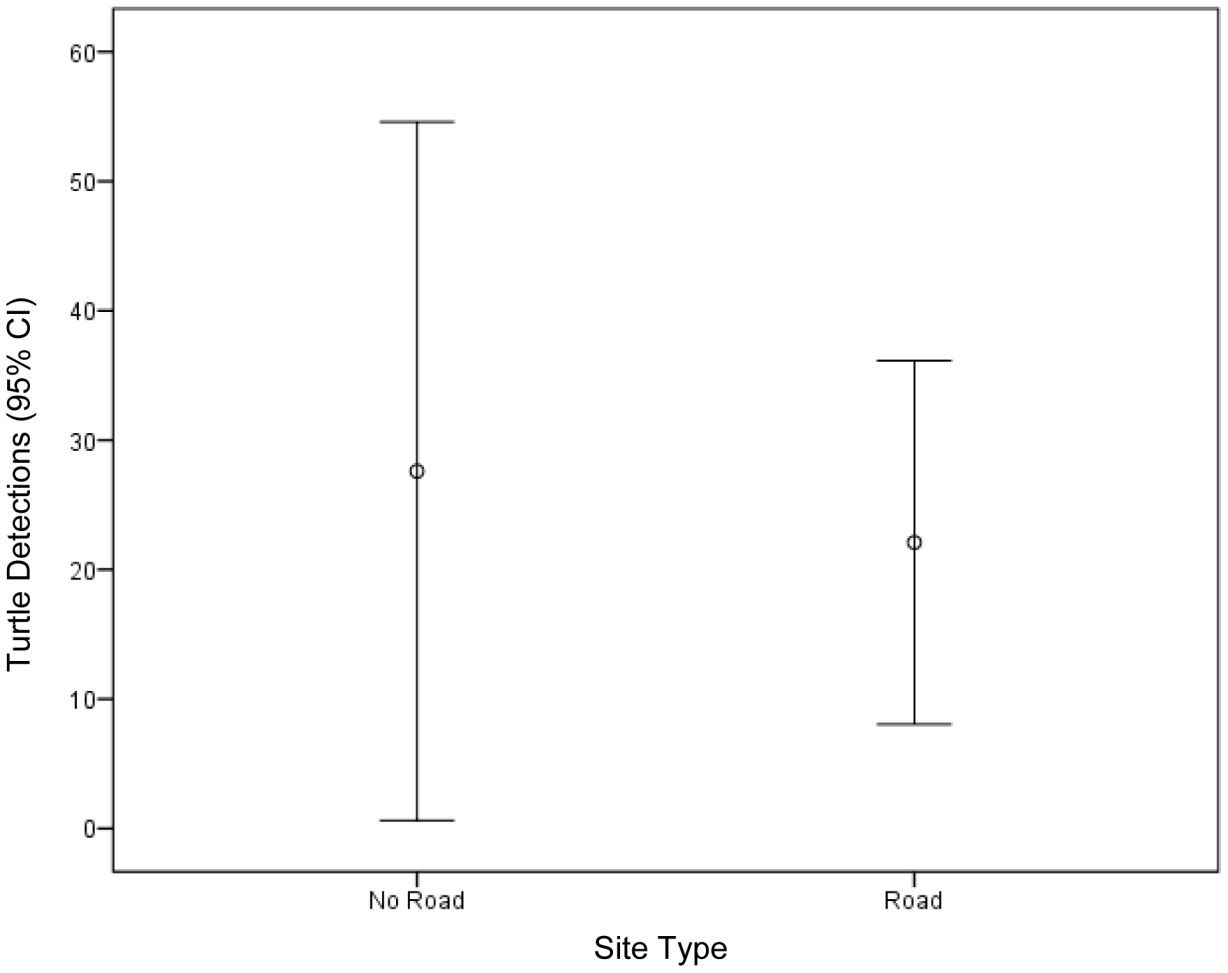
Mean Turtle Detections at Road and No Road sites. Mean (untransformed) Turtle Detections and 95% confidence intervals (CI) at Road and No Road sites. Note, there were two ponds at the No Road sites where no turtles were observed and one pond where 121 turtles were observed (Table S2 and S3).

Mean (untransformed) Turtle Detections at Road and No Road sites were 22.1 (±6.2 SE) and 27.6 (±11.9 SE) respectively. Ninety-five percent confidence intervals (CI) completely overlap between the two site types (Figure 3). Both sites where no turtles were detected were No Road sites. The slightly higher mean abundance for the No Road sites was due entirely to a single No Road site that had the largest number of turtle detections of any site (121) (Table S2 and S3).

**Figure 4:**
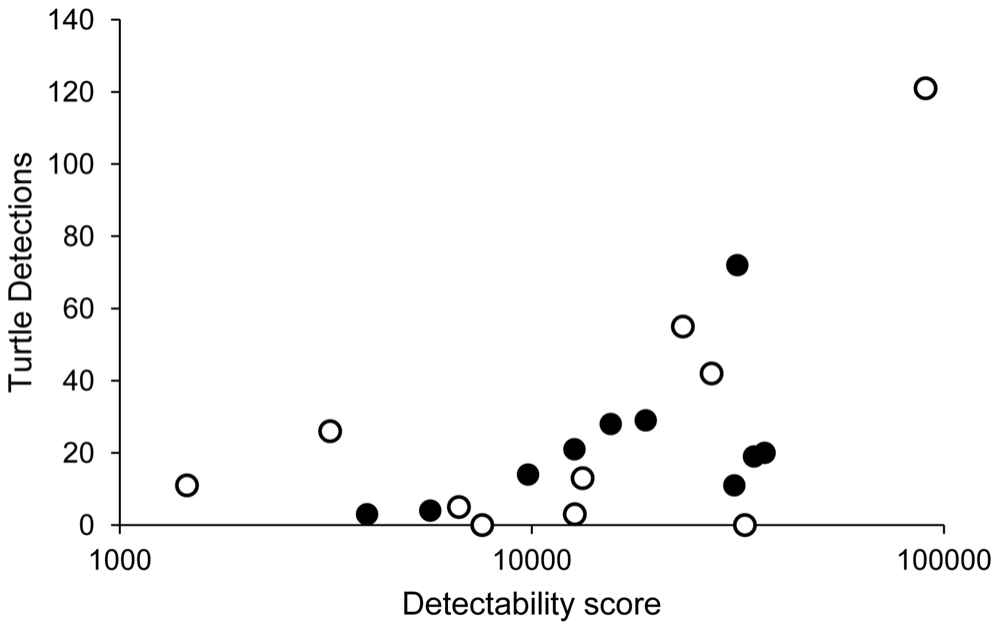
Turtle Detections vs. Detectability score. The number of Turtle Detections at Road (•) and No Road (Ο) sites against Detectability score. Turtle Detections included all turtle sightings and captures that were affected by detectability. The Detectability score was created as the product of pond perimeter (m) and pond visibility (average visible depth (cm)). Note, for analyses we log transformed Turtle Detections (log [Turtle Detections +1]) (the response variable), but Detectability score (the predictor) was not transformed. The raw data for the Detectability score were plotted on a log scale for this figure.

None of the potential confounding local site or landscape variables differed significantly between the Road and No Road sites (Table S4); therefore, they were not included in further analyses. Detectability scores did not differ significantly between Road and No Road sites (*t* = −0.199, *df*  = 18, *p* = 0.845). There was a significant positive relationship between Detectability score and the number of Turtle Detections (Figure 4; *β* = 0.474; *F* = 5.229, *df*  = 1, 18, *p* = 0.035, *R^2^* = 0.225), with Detectability score accounting for just under 23% of the variation in the number of Turtle Detections. Therefore, we included Detectability in further analyses of Turtle Detections to account for variability due to detectability.

**Figure 5:**
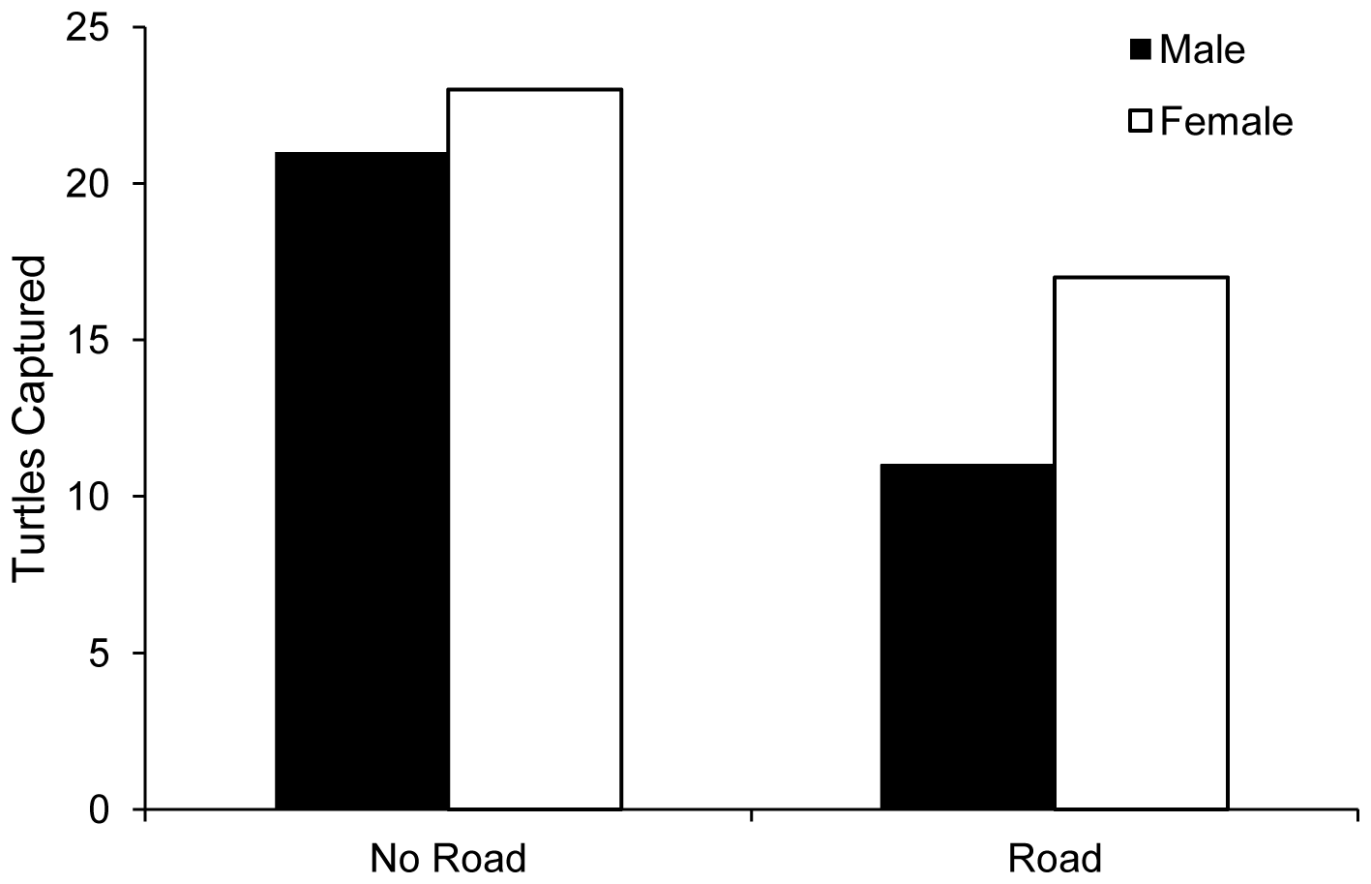
Numbers of male and female turtles captured at Road and No Road sites.

**Figure 6:**
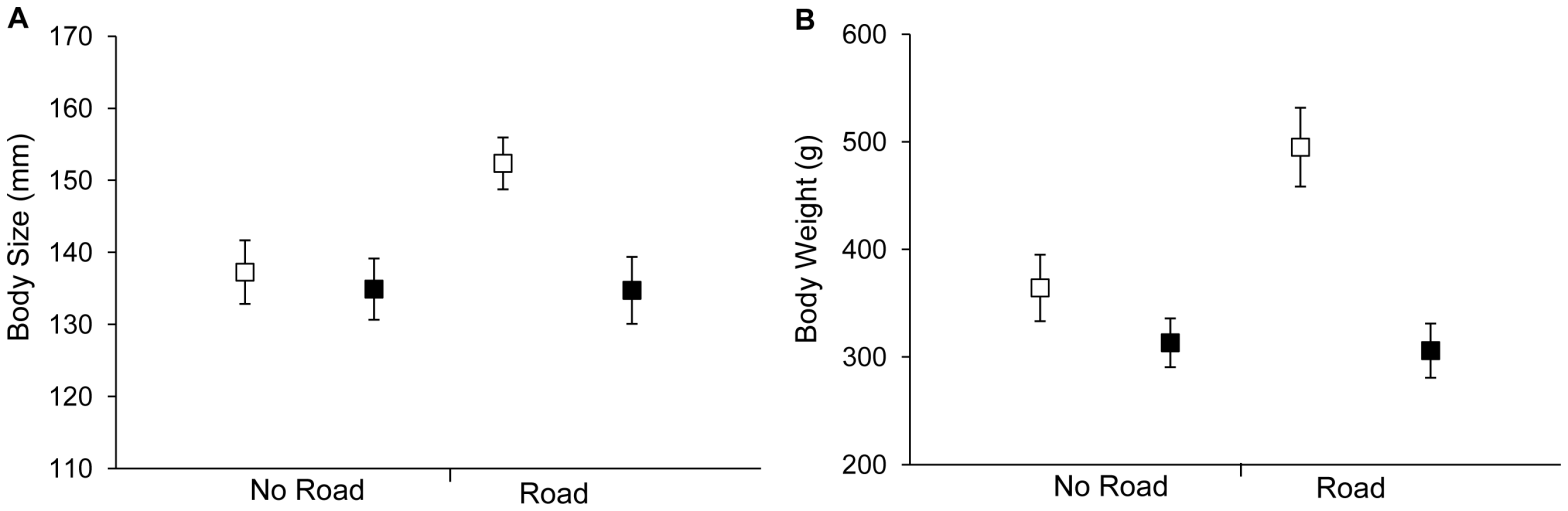
Carapace length and body weight of captured turtles. Panel (A) shows mean body size of captured females (□) and males (▪), measured as straight line carapace length in mm, at No Road and Road sites. Panel (B) shows mean body weight in g of captured females (□) and males (▪), at No Road and Road sites.

The multiple regressions of Turtle Detections on Detectability and each of (a) Site Type (Road vs. No Road), (b) road density, and (c) total traffic volume, revealed no significant effects of road-related predictors on turtle relative abundance (Table 1). Contrary to expectations, the proportion of captured turtles that were male was lower in No Road than Road sites, but this difference was not statistically significant (*Pearson's X^2^*_(n = 72)_  = 0.494, *df*  = 1, *p* = 0.482; Figure 5). Males showed no significant difference in body size (measured as straight-line carapace length) or weight between Road and No Road sites (body size: *t* = 0.026, *df*  = 30, *p* = 0.979; weight: *t* = 0.202, *df*  = 30, *p* = 0.841; Figure 6). In contrast, and opposite to our prediction, females were significantly larger and heavier at Road sites than at No Road sites (body size: *t* = −2.737, *df*  = 38, *p* = 0.009; weight: *t* = −2.649, *df*  = 37.92, *p* = 0.012; Figure 6).

**Table 1:** Turtle relative abundance vs. detectability and road predictors.

	β Detectability	β Site type	β Road density	β Total AADT	R^2^	F	df	p
(a)	0.483	0.189	-	-	0.261	3.001	2, 17	0.077
(b)	0.473	-	−0.037	-	0.236	2.489	2, 17	0.113
(c)	0.476	-	-	0.009	0.225	2.470	2, 17	0.114

Model summaries of the relationship between turtle relative abundance (log[Turtle Detections +1]) and (a) Detectability score and Site Type (Road vs. No Road), (b) Detectability score and Road Density (m/km^2^), and (c) Detectability score and total traffic volume (Total AADT). Turtle Detections included all turtle sightings and captures that were affected by detectability. The Detectability score was created as the product of pond perimeter and pond visibility (average visible depth). Both road density and total AADT were measured within 300 m of the pond.

## Discussion

The purpose of this study was to conduct a strong test of the prediction that road mortality reduces painted turtle population abundance. We found no statistically significant difference in turtle relative abundance between Road and No Road sites, suggesting that the effects of road mortality, which we assume to be high at ponds near high traffic roads, may not translate into significant effects on population abundance. There are two possible reasons for this: (1) the predicted negative effect of road mortality on turtle populations was present but we were not able to detect it in the current study, or (2) although many turtles are killed on roads each year, there is no overall negative effect on turtle populations due to compensatory factors. We discuss each of these in turn.

If road mortality does negatively affect painted turtle populations, then why were we not able to detect a significant effect here? Some studies have suggested that the negative effects of road mortality may be hard to detect in areas where roads are relatively new due to turtle longevity and delayed sexual maturity, which may cause a time lag in detecting an effect on the population [9], [22]. It is, however, unlikely that this was an issue in this study as we selected only ponds and adjacent roads that were several decades old, presumably allowing sufficient time for road mortality to affect the populations at these sites (i.e., the average age of the high traffic roads close to the Road site ponds was 55 years (± SE  = 4.8 years) and 46 years (±0.9 years) at the No Road sites). Confounding variables, masking a negative effect of roads on turtle populations, are another potential reason that we did not observe a road effect. Past studies have found that turtle abundance was correlated with the amount of forest in the landscape surrounding the wetland or pond [23], [38] and the amount of emergent vegetation in and around the water body [18]. However, we controlled for these variables and others in our study design by selecting sites that did not differ significantly in any of the measured local and landscape variables (Table S4). Thus, it is unlikely that they masked an effect of roads on turtle relative abundance. In addition, none of these variables was significantly related to turtle relative abundance (Table S5), indicating it is unlikely that the variance explained by them obscured our ability to detect a difference in abundance between Road and No Road sites.

Another possible reason that we did not detect a road effect on turtle relative abundance is the difficulty of accurately estimating turtle abundance due to detectability issues. Mark-recapture studies of a single wetland or a small group of ponds in close proximity can arrive at relatively good population estimates after several years of intensive sampling (e.g., [47]). However, evaluating a small number of turtle populations is not sufficient when testing a prediction such as the one we tested. Therefore, our approach was to measure the variables likely to cause variation in detectability among ponds and to control for detectability in the analysis. Turtle abundance increased with our Detectability score, and turtle abundance did not differ significantly between Road and No Road sites after accounting for Detectability. Note, our results did not qualitatively change if we regressed Turtle Detections per pond perimeter searched (m) on pond visibility and Site Type (Road vs. No Road) rather than combining pond perimeter and pond visibility into a single Detectability measure and regressing Turtle Detections on Detectability and Site Type. It remains possible that error in relative population estimates played a role in our inability to detect a difference in relative abundance between the Road and No Road sites. For example, our Turtle Detections metric could be composed of repeated sightings of the same individual at a given site on different survey dates. This may present a problem if for example individual turtles at the Road sites were more likely to be repeatedly observed through visual surveys than turtles at the No Road sites. If that were true, our Turtle Detections metric would be an overestimate of the relative turtle abundance at the Road sites, potentially masking a negative effect of roads on turtle populations. However, given our survey protocol, turtles should have been equally likely to be repeatedly sighted at both site types, essentially resulting in a true indicator of relative abundance among sites. Given that we successfully controlled for detectability, consistently followed the same sampling protocol at all survey sites, and given our large sample size relative to other studies (two pond types, with 10 in each category), we conclude that our results indicate either no effect or only a very weak effect of roads on relative abundance of painted turtles.

Overall then, our results suggest that there is either no road effect or only a very weak road effect on painted turtle populations in our study. Despite many studies having suggested that roads should have a strong negative effect on turtle populations, our results are consistent with a previous modeling study that predicted no road effect on small-bodied turtle populations. Gibbs and Shriver [6] investigated the effects of roads on turtle population persistence by simulating turtle movements in urban and rural landscapes of varying road density and traffic volumes. They modelled annual road-associated mortality in three groups of turtles (1- land turtles such as box turtles (*Terrapene*), 2- small-bodied pond turtles such as painted turtles (*Chrysemys picta*), and 3 - large-bodied pond turtles such as snapping turtles (*Chelydra serpentina*)) and found that roads had the potential to decrease population size in land and large-bodied pond turtles, but no such effect was predicted for small-bodied pond turtles. Our finding of no road effect on painted turtle populations provides empirical support for Gibbs and Shiver's [6] model prediction for smaller-bodied turtles.

A possible reason for our finding of no road effect or only a very weak road effect on painted turtle populations is that very high traffic roads may simply act as movement barriers without causing excessive mortality. In other words, turtles may not even attempt to cross the roads due to the constant heavy volume of traffic. Road avoidance behaviour has been reported in Blanding's turtles (*Emydoidea blandingii*) close to our study location. Proulx *et al*. [48] found that radio-tracked Blanding's turtles in Québec crossed roads significantly less often than predicted. Interestingly, an individual's tendency to cross roads was not influenced by its sex, by the road surface (unpaved or paved), or by roads being open to vehicle traffic or not [48]. Road avoidance behaviour in painted turtles has not been empirically quantified as of yet but studies have shown that they frequently cross roads (e.g., [23], [49]). We therefore suggest it is unlikely that the lack of road effect on painted turtle abundance was due to turtle avoidance of roads.

The lack of road effect we observed might be explained by turtles remaining very close to the pond edge, if there were more than enough nesting sites available immediately adjacent to the ponds. In order for there to be a negative effect of roads due to road mortality, turtles must be compelled to cross or move along roads. Road mortality may not be an issue if turtles have no reason to leave the immediate vicinity of the pond. Previous studies have shown that turtle abundance is positively related to the amount of nesting habitat found in the surrounding landscape [18] and that most terrestrial movement by female turtles are to find suitable nesting sites [10], [19]. Baldwin *et al*. [23] found that the mean distance traveled by nesting female painted turtles in southeastern New Hampshire was negatively correlated with the abundance of nesting habitat near pond edges. Although we measured some local habitat characteristics surrounding the pond that may be indicative of nesting habitat availability (i.e., percent area within 5-m radius of pond edge covered by grass or open ground), more accurate measures of suitable nesting habitat (e.g., soil drainage and open canopy; [4]) would be required to address whether this could be the reason we did not detect an effect of roads on turtle relative abundance. However, we designed our study specifically to minimize the distances between the Road ponds and the neighbouring high traffic roads (mean distance to high traffic road  = 65 m±10 m (SE)) so any suitable nesting habitat along the roads in our study might be reasonably considered to be part of the near-pond nesting habitat.

Another reason for a lack of effect of roads on painted turtle abundance could be that rapid evolution has reduced road-crossing tendency in populations near roads. If road mortality is high, this could lead to rapid selection against individuals undertaking movements over roads. Janzen and Morjan [50] found that certain aspects of painted turtle nesting behaviour may indeed be subject to microevolution. Temple [11] found that significantly more females were nesting away from ecological edges, where nest predation was higher, than would be expected if they were nesting randomly within the landscape, suggesting that selection may work in favour of females that nest far from ecological edges. It is therefore possible that selective pressures from road mortality have favoured females that nest closer to ponds at sites close to high traffic roads, eliminating or greatly reducing the effect of road mortality on turtle abundance.

Another possible reason for our finding of no road effect or only a very weak road effect on painted turtle populations is that roadside painted turtle populations may remain relatively high because of population supplementation from emigrants originating from ponds distant from roads. When selecting our study sites, we had a difficult time finding ponds in our study area that were completely isolated from any other obvious water. We selected only ponds that were a minimum distance of 250 m from other water bodies (mean distance to nearest body of water  = 372 m±27 m (SE); range  = 250–690). This distance resulted from a trade-off in which we attempted to maximize both pond isolation and the number of study sites sampled. While we based the 250 m minimum on previous studies reporting that most painted turtle movements occur within 300 m of the pond edge [10], [23], [32]–[34], we acknowledge that painted turtles have been reported to move as much as 3.3 km between wetlands [51]. Therefore, we cannot rule out the possibility that roadside wetlands could be an ecological trap and may even maintain relatively high population densities if there are ample source populations distant from roads. This may have reduced the apparent effect of roads on turtle populations.

It is also possible that, while road mortality alone could cause a reduction in turtle abundance, this mortality is compensated by positive effects of roads on painted turtle populations. For example, predation may be lower on nests near roads. Turtles experience their highest mortality rate as eggs or young in the nest, much of this being due to nest predation by species such as racoons (*Procyon lotor*) and foxes (*Vulpes vulpes*), among others [1], [10], [25], [42], [52], [53]. Studies have found reduced predation of turtle nests farther from the pond edge [10], [23], [54], [55] and closer to road edges [56]. For example, Langen [49] found that the risk of nest predation was significantly lower near a high-traffic highway than at nesting sites away from public roads. In addition, numerous studies have shown that predator species are particularly susceptible to the negative impacts of roads (reviewed in Fahrig and Rytwinski [57], Rytwinski and Fahrig [58]) and theoretical work suggests that this can result in indirect positive effects of roads on their prey [59]. It is therefore possible that the negative effect of road mortality on painted turtles is outweighed by the positive effect of predation release on turtle eggs and young.

We did not find the predicted increase in male (and decrease in female) representation at Road sites relative to No Road sites. If anything, the data suggest the opposite, although the difference was not significant (Figure 5). There are two common arguments in the literature as to which sex should be more vulnerable to road mortality. It has been argued that more vagile individuals should encounter roads more frequently and thus be more vulnerable to road mortality [6], [57], [59]–[62]. Studies of movement patterns of turtles suggest that males are the more vagile sex, dispersing more frequently and farther than females [33], [63], [64]. It has been suggested that male turtles should, therefore, disperse more frequently and thus experience greater road mortality [26], [29]. In contrast, it has been argued that the annual nesting migrations, the pre-nesting overland excursions, and the attraction to roadside nesting sites of female turtles place them at a greater risk of road mortality (e.g., [10], [22], [26]). From these contrasting arguments then it seems unclear whether turtle populations near roads should be female or male-biased. Much of the road ecology studies to date support the latter, as sex ratios of turtle populations near roads are often biased towards males [18]–[20], [22]. In addition, studies have found proportionally more female turtles (dead or alive) on roads than males (reviewed in Steen *et al*. [26]). Therefore, the evidence to date suggests that female turtles should be more vulnerable to road mortality, and thus, sex ratios should be more skewed towards males in ponds near roads.

Why then did we not find this pattern? There are two other studies that found no difference in sex ratios between turtle populations in disturbed areas or areas of higher vehicular traffic versus more natural areas or areas of lower vehicular traffic ([39]: eastern long-necked turtle (*Chelodina longicollis*); [29]: desert tortoise (*Gopherus agassizii*)). Although these studies provide some corroboration for our finding of no difference in sex ratios of painted turtle populations in ponds near roads, suggesting that both sexes are susceptible to road mortality, it remains unclear as to why we observed a slight female-biased sex ratio in turtle populations near roads. If the most likely explanation for the lack of effect of roads on painted turtle abundance is reduced predation on eggs and young of roadside nests, we should still expect to see a male-biased sex ratio (even a slight bias) in ponds near roads because turtles nest along roads and previous studies suggest that females are the more vulnerable sex to road mortality. Given that sex determination in turtles is temperature dependent, with higher temperatures producing mostly female hatchlings, it is possible that warmer nest temperatures near roads could produce more females thus resulting in the slight female-biased sex ratio observed in ponds near roads in our study location. Langen [49] reported that roadside nests were warmer and more variable in temperature than nests away from paved roads, with temperature maxima at times as much as 6°C higher at the roadside than other sites. The sex ratio of hatchlings from these nests were not provided; however, it was concluded that differences in sex ratios between nests along roadsides and other sites are likely given the magnitude of the difference in temperature [49]. Therefore it is possible that warmer nest temperatures near roads could cause sex ratios in painted turtle populations to be slightly female-biased. This hypothesis remains speculative.

We did not find the predicted decrease in female body size at Road sites relative to No Road sites. In fact, females were significantly larger and heavier in ponds close to high traffic roads than in ponds far from high traffic roads. Males did not follow this trend. Similar to our result, Roe *et al*. [39], comparing eastern long-necked turtles (*Chelodina longicollis*) (a medium-sized freshwater turtle) in a suburban landscape vs. an adjacent nature reserve, found larger turtles in the disturbed suburban areas. However, they found this to be true for both males and females while we found it only for females. Roe *et al*. [39] also found that adult turtles in the suburban population exhibited much higher growth rates than those found in the nearby nature reserve. We find this explanation unlikely in our study. The fact that our Road and No Road sites did not differ significantly in local or landscape scale habitat variables and that the average body size increase was only observed in females do not support the hypothesis that the Road sites generally favour higher turtle growth rates.

It is possible that natural selection favours larger females in areas near roads because nest construction is more difficult on road shoulders than in more natural areas. Refsnider and Linck [65] found that Blanding's turtles that nested in or along gravel road shoulders or trails generally made more nesting attempts, and spent more time excavating the nest cavities, than turtles that nested elsewhere. If nest construction along roads is more difficult than in more natural habitat types, this should favour larger and stronger females in roaded areas. However, to our knowledge no other studies have reported larger females at ponds near roads, so this explanation remains highly speculative.

The fact that females were larger at Road sites than at No Road sites (whatever the reason) provides an additional possible explanation for our result of no road effect on painted turtle relative abundance. Body size in many reptiles, including the painted turtle, correlates strongly with fecundity [66]. If the larger females at Road sites produce larger clutches than those at No Road sites, this may compensate for the negative effect of road mortality, such that there is no net negative effect of roads on the turtle populations. Supporting this hypothesis, Langen [49] found more hatchlings in roadside nests than in nests away from roads and that hatchlings from roadside nests were larger and heavier than hatchlings from nests elsewhere. While these observations are preliminary and suffer from small sample sizes, they do suggest that larger females laying larger clutches near roads may counter-balance the negative effects of road mortality on painted turtle populations. However, since we are not aware of any other studies reporting larger females at ponds near roads, this explanation remains speculative.

Overall, we cannot strongly conclude in favour of a single explanation accounting for all of our results. Nevertheless, we suggest the most likely explanation for our result on abundance relates to compensatory factors counter-balancing the effects of road mortality. These could include lower predation on eggs and young in nests near roads than in nests away from roads and larger clutch sizes in nests near roads due to larger females at these sites.

Our study highlights a few unanswered questions. First, although road mortality is suggested to be a leading cause of turtle population declines, there is currently very limited data demonstrating that freshwater turtle population abundance or distribution is negatively affected by roads. To effectively mitigate road impacts, it is necessary to know which species are most vulnerable to roads and in what way(s) roads impact those species (e.g., through road mortality, loss/alteration of habitat, or habitat fragmentation). Our results suggest that not all turtle species are negatively affected by roads. More studies quantifying road effects on turtle populations are needed. Second, more research is needed on the nesting behaviour of females near roads and the effects of roadside nesting on the turtle populations. For example, are females that choose roadside nest sites actually attracted to these or are these sites their only available option? How are hatchling success and sex ratios affected by roadside nesting? How do the higher temperatures or daily fluctuations in temperatures at roadside nests affect hatchling viability?

In conclusion, our results suggest that high traffic roads, and thus presumably high road mortality, do not negatively affect painted turtle populations. However, it would not be appropriate to extrapolate this result to other turtle species. Gibbs and Shriver [6] predicted larger-bodied pond turtles and terrestrial turtles could show population declines as a result of road mortality. Furthermore, negative effects of roads have been reported on tortoise populations [28], [29], and a recent study [30] estimated that per capita road mortality of female diamondback terrapins (*Malaclemys terrapin*) was high enough to cause a population decline.

Even for small aquatic turtles, there are likely particular situations in which road mortality is so high that it must affect population persistence. For example, when drought occurs in Lake Jackson, Florida, turtle populations of several species - some of which are small aquatic or semi-aquatic turtles (e.g., *Deirochelys reticularia, Kinosternon subrubrum, Sternotherus odoratus*) - undergo mass migration into the larger, permanently wet portion of Lake Jackson, with more than 1200 turtles attempting to cross Highway 27 per km per year [67]. Since each such migration kills about 98% of the entire Little Lake Jackson turtle populations, population effects seem certain even on the smaller species. In situations such as the Lake Jackson turtle populations, mitigation of road mortality, by fencing or other means of keeping turtles off the road, and a way for the turtles to pass under the road to the lake is clearly necessary. In contrast, in situations where turtles are not “forced” to cross a road, such as in our study, road mitigation for painted turtles may not be necessary.

## Supporting Information

Figure S1 **Summary of the sampling protocol.** Ponds were paired (pond 1 =  white box, pond 2 =  grey box) and sampled twice daily for three consecutive days twice during the summer (totalling six sampling days at each pond). Sampling days were divided into four periods, Morning 1 (M1), Morning 2 (M2), Afternoon 1 (A1), and Afternoon 2 (A2), such that each of the ponds within a sampling pair was visited once in the morning and once in the afternoon each sampling day. The order of visits alternated each day.
(DOC)

Table S1 **Study site locations.** Global positioning system (GPS) coordinates of the Road (R#) and No Road (NR#) sites. Most (18) study sites were located on privately owned land, and two (R2 and R5) were located on crown land.
(DOCX)

Table S2 **The total number of turtle observations between 1 June and 28 August 2011.** “Turtle Sightings” represented all turtles seen searching along the perimeter (<3 m from shore) of the pond either by canoe or by foot. “Turtles Captured” included turtles that were captured either by dipnet, by hand, or by hoopnet, and individually marked. “Turtle Detections” represented sightings and captures that were affected by detectability and included all turtle sightings and turtles captured either by dipnet or by hand. “Turtle Observations” represented sightings and total turtle captures.
(DOC)

Table S3 **All turtles captured by dipnet or hoopnet between 1 June and 28 August 2011.** Sex was determined using secondary sex characteristics: the male cloacal opening extends past the posterior edge of the carapace while the female cloacal opening does not. Males also have much longer foreclaws. We used the carapace length of the smallest identifiable male we sampled as the minimum size for assigning gender.
(DOC)

Table S4 **Results of t-tests comparing local and landscape variables at Road and No Road sites.** Variables (a)–(d) weremeasured within a 5-m radius of the pond edge, variables (e)–(j) were measured at the surface of each pond, and variables (k)–(m) were measured within a 300-m radius of each pond.
(DOC)

Table S5 **Model summaries of simple linear regressions of the relationship between turtle relative abundance (log[Turtle Detections +1]) and predictor variables.** Predictor variables (a)–(d) were measured within a 5-m radius of the pond edge, variables (e)–(j) were measured at the surface of each pond, and variables (k)–(m) were measured within a 300-m radius of each pond.
(DOC)
