# Supplementary material for: Do Roads Reduce Painted Turtle (Chrysemys picta) Populations?
Source: PLoS One. 2014 May 23;9(5):e98414. doi: 10.1371/journal.pone.0098414 (PMC4032323; doi:10.1371/journal.pone.0098414)
Supplement: Figure S1 — Summary of the sampling protocol. Ponds were paired (pond 1 = white box, pond 2 = grey box) and sampled twice daily for three consecutive days twice during the summer (totalling six sampling days at each pond). Sampling days were divided into four periods, Morning 1 (M1), Morning 2 (M2), Afternoon 1 (A1), and Afternoon 2 (A2), such that each of the ponds within a sampling pair was visited once in the morning and once in the afternoon each sampling day. The order of visits alternated each day. (DOC) [file pone.0098414.s001.doc]

**Figure S1. Summary of the sampling protocol.** Ponds were paired (pond 1 = white box, pond 2 = grey box) and sampled twice daily for three consecutive days twice during the summer (totalling six sampling days at each pond). Sampling days were divided into four periods, Morning 1 (M1), Morning 2 (M2), Afternoon 1 (A1) and Afternoon 2 (A2), such that each of the ponds within a sampling pair was visited once in the morning and once in the afternoon each sampling day. The order of visits alternated each day.

**Pond 2**

*(Perimeter search & hoopnet set-up)*

**Pond 2**

*(Perimeter search & hoopnet check)*

**Pond 1**

*(Perimeter search & hoopnet set-up)*

**Pond 1**

*(Perimeter search & hoopnet check)*

**Pond 2**

*(Perimeter search & hoopnet check)*

**Pond 1**

*(Perimeter search & hoopnet check)*

**Pond 1**

*(Perimeter search & hoopnet check)*

**Pond 2**

*(Perimeter search & hoopnet check)*

**Pond 2**

*(Perimeter search & hoopnet removal)*

**Pond 1**

*(Perimeter search & hoopnet removal)*

**Pond 2**

*(Perimeter search)*

**Pond 1**

*(Perimeter search)*

Day 4

Day 5

Day 6

**Pond 1**

*(Perimeter search & hoopnet set-up)*

**Pond 1**

*(Perimeter search & hoopnet check)*

**Pond 2**

*(Perimeter search & hoopnet set-up)*

**Pond 2**

*(Perimeter search & hoopnet check)*

**Pond 1**

*(Perimeter search & hoopnet check)*

**Pond 2**

*(Perimeter search & hoopnet check)*

**Pond 2**

*(Perimeter search & hoopnet check)*

**Pond 1**

*(Perimeter search & hoopnet check)*

**Pond 1**

*(Perimeter search & hoopnet removal)*

**Pond 2**

*(Perimeter search & hoopnet removal)*

**Pond 1**

*(Perimeter search)*

**Pond 2**

*(Perimeter search)*

Day 1

Day 2

Day 3

M1

(08:00-10:20)

M2

(09:30-12:30)

A1

(12:30-14:30)

A2

(13:15-16:45)

(Approximately 6 weeks passes between sampling Day 3 and Day 4)
