# Supplementary material for: Do Roads Reduce Painted Turtle (Chrysemys picta) Populations?
Source: PLoS One. 2014 May 23;9(5):e98414. doi: 10.1371/journal.pone.0098414 (PMC4032323; doi:10.1371/journal.pone.0098414)
Supplement: Table S1 — Study site locations. Global positioning system (GPS) coordinates of the Road (R#) and No Road (NR#) sites. Most (18) study sites were located on privately owned land, and two (R2 and R5) were located on crown land. (DOCX) [file pone.0098414.s002.docx]

**Table S1. Study site locations.** Global positioning system (GPS) coordinates of the Road (R#) and No Road (NR#) sites. Most (18) study sites were located on privately owned land, and two (R2 and R5) were located on crown land.

| **Site Code** | **GPS Coordinates*** |
| --- | --- |
| R1 | 45°26'2.28"N 75°35'54.79"W |
| R2 | 45°1'24.55"N 74°57'35.71"W |
| R3 | 45°18'28.50"N 75°59'33.35"W |
| R4 | 44^o^89'42.94"N 75°25'76.78"W |
| R5 | 44°51'18.60"N 75°19'50.85"W |
| R6 | 45°4'32.07"N 76°14'43.08"W |
| R7 | 44°56'2.30"N 76°14'30.93"W |
| R8 | 45°20'16.56"N 75°21'48.13"W |
| R9 | 45°17'38.60"N 76°5'0.25"W |
| R10 | 45°12'2.45"N 76°2'19.25"W |
| NR1 | 45°23'30.80"N 76°38'20.88"W |
| NR2 | 45°26'26.59"N 76°40'26.30"W |
| NR3 | 45°23'0.48"N 75°18'22.74"W |
| NR4 | 45°3'14.12"N 74°55'3.07"W |
| NR5 | 45°21'44.24"N 75°59'20.07"W |
| NR6 | 45°09'38.07"N 75°16'03.35"W |
| NR7 | 45°54'23.07"N 75°09'42.13"W |
| NR8 | 45°33'12.00"N 75°15'0.91"W |
| NR9 | 45°6'4.56"N 74°56'12.96"W |
| NR10 | 45°10'12.74"N 74°55'0.20"W |

* Contact A. Dorland for further information on study site location information.
