# Supplementary material for: Do Roads Reduce Painted Turtle (Chrysemys picta) Populations?
Source: PLoS One. 2014 May 23;9(5):e98414. doi: 10.1371/journal.pone.0098414 (PMC4032323; doi:10.1371/journal.pone.0098414)
Supplement: Table S2 — The total number of turtle observations between 1 June and 28 August 2011. “Turtle Sightings” represented all turtles seen searching along the perimeter (<3 m from shore) of the pond either by canoe or by foot. “Turtles Captured” included turtles that were captured either by dipnet, by hand, or by hoopnet, and individually marked. “Turtle Detections” represented sightings and captures that were affected by detectability and included all turtle sightings and turtles captured either by dipnet or by hand. “Turtle Observations” represented sightings and total turtle captures. (DOC) [file pone.0098414.s003.doc]

**Table S2. The total number of turtle observations between 1 June and 28 August 2011**. “Turtle Sightings” represents all turtles seen searching along the perimeter (< 3 m from shore) of the pond either by canoe or by foot. “Turtles Captured” includes turtles that were captured either by dipnet, by hand, or by hoopnet, and individually marked. “Turtle Detections” represents sightings and captures that were affected by detectability and includes all turtle sightings and turtles captured either by dipnet or by hand. “Turtle Observations” represents sightings and total turtle captures.

| **Site type** | **Site** | **Turtle Sightings** | **Turtles Captured** | | | **Turtle Detections** | **Turtle Observations** |
| --- | --- | --- | --- | --- | --- | --- | --- |
|  |  |  | *Dipnet+Hand* | *Hoopnet* | *Total* |  |  |
| Road | R1 | 4 | 0 | 3 | 3 | 4 | 7 |
|  | R2 | 21 | 0 | 4 | 4 | 21 | 25 |
|  | R3 | 69 | 3 | 2 | 5 | 72 | 74 |
|  | R4 | 14 | 0 | 2 | 2 | 14 | 16 |
|  | R5 | 19 | 0 | 4 | 4 | 19 | 23 |
|  | R6 | 3 | 0 | 1 | 1 | 3 | 4 |
|  | R7 | 26 | 2 | 5 | 7 | 28 | 33 |
|  | R8 | 29 | 0 | 2 | 2 | 29 | 31 |
|  | R9 | 20 | 0 | 2 | 2 | 20 | 22 |
|  | R10 | 11 | 0 | 0 | 0 | 11 | 11 |
|  | **Total** | **216** | **5** | **25** | **30** | **221** | **246** |
| No Road | NR1 | 113 | 8 | 10 | 18 | 121 | 131 |
|  | NR2 | 55 | 0 | 7 | 7 | 55 | 62 |
|  | NR3 | 11 | 0 | 6 | 6 | 11 | 17 |
|  | NR4 | 3 | 0 | 4 | 4 | 3 | 7 |
|  | NR5 | 0 | 0 | 0 | 0 | 0 | 0 |
|  | NR6 | 13 | 0 | 2 | 2 | 13 | 15 |
|  | NR7 | 41 | 1 | 1 | 2 | 42 | 43 |
|  | NR8 | 5 | 0 | 0 | 0 | 5 | 5 |
|  | NR9 | 21 | 5 | 2 | 7 | 26 | 28 |
|  | NR10 | 0 | 0 | 0 | 0 | 0 | 0 |
|  | **Total** | **262** | **14** | **32** | **46** | **276** | **308** |
| **Grand Total** | | **478** | **19** | **57** | **76** | **497** | **554** |
