# Supplementary material for: Do Roads Reduce Painted Turtle (Chrysemys picta) Populations?
Source: PLoS One. 2014 May 23;9(5):e98414. doi: 10.1371/journal.pone.0098414 (PMC4032323; doi:10.1371/journal.pone.0098414)
Supplement: Table S3 — All turtles captured by dipnet or hoopnet between 1 June and 28 August 2011. Sex was determined using secondary sex characteristics: the male cloacal opening extends past the posterior edge of the carapace while the female cloacal opening does not. Males also have much longer foreclaws. We used the carapace length of the smallest identifiable male we sampled as the minimum size for assigning gender. (DOC) [file pone.0098414.s004.doc]

**Table S3. All turtles captured by dipnet or hoopnet between 1 June and 28 August 2011.** Sex was determined using secondary sex characteristics: the male cloacal opening extends past the posterior edge of the carapace while the female cloacal opening does not. Males also have much longer foreclaws. We used the carapace length of the smallest identifiable male we sampled as the minimum size for assigning gender.

| **Site Type** | **Site** | **Females** | | | **Males** | | | **Juveniles** | | |
| --- | --- | --- | --- | --- | --- | --- | --- | --- | --- | --- |
|  |  | *Dipnet* | *Hoopnet* | *Total* | *Dipnet* | *Hoopnet* | *Total* | *Dipnet* | *Hoopnet* | *Total* |
| Road | R1 | 0 | 2 | 2 | 0 | 1 | 1 | 0 | 0 | 0 |
|  | R2 | 0 | 3 | 3 | 0 | 1 | 1 | 0 | 0 | 0 |
|  | R3 | 2 | 1 | 3 | 0 | 1 | 1 | 1 | 0 | 1 |
|  | R4 | 0 | 1 | 1 | 0 | 0 | 0 | 0 | 1 | 1 |
|  | R5 | 0 | 1 | 1 | 0 | 3 | 3 | 0 | 0 | 0 |
|  | R6 | 0 | 1 | 1 | 0 | 0 | 0 | 0 | 0 | 0 |
|  | R7 | 2 | 1 | 3 | 0 | 4 | 4 | 0 | 0 | 0 |
|  | R8 | 0 | 2 | 2 | 0 | 0 | 0 | 0 | 0 | 0 |
|  | R9 | 0 | 1 | 1 | 0 | 1 | 1 | 0 | 0 | 0 |
|  | R10 | 0 | 0 | 0 | 0 | 0 | 0 | 0 | 0 | 0 |
|  | **Total** | **4** | **13** | **17** | **0** | **11** | **11** | **1** | **1** | **2** |
| No Road | NR1 | 7 | 4 | 11 | 0 | 5 | 5 | 1 | 1 | 2 |
|  | NR2 | 1 | 1 | 2 | 0 | 5 | 5 | 0 | 0 | 0 |
|  | NR3 | 0 | 2 | 2 | 0 | 4 | 4 | 0 | 0 | 0 |
|  | NR4 | 0 | 3 | 3 | 0 | 1 | 1 | 0 | 0 | 0 |
|  | NR5 | 0 | 0 | 0 | 0 | 0 | 0 | 0 | 0 | 0 |
|  | NR6 | 0 | 2 | 2 | 0 | 0 | 0 | 0 | 0 | 0 |
|  | NR7 | 1 | 0 | 1 | 0 | 1 | 1 | 0 | 0 | 0 |
|  | NR8 | 0 | 0 | 0 | 0 | 0 | 0 | 0 | 0 | 0 |
|  | NR9 | 2 | 0 | 2 | 3 | 2 | 5 | 0 | 0 | 0 |
|  | NR10 | 0 | 0 | 0 | 0 | 0 | 0 | 0 | 0 | 0 |
|  | **Total** | **11** | **12** | **23** | **3** | **18** | **21** | **1** | **1** | **2** |
| **Grand Total** | | **15** | **25** | **40** | **3** | **29** | **32** | **2** | **2** | **4** |
