# Supplementary material for: Do Roads Reduce Painted Turtle (Chrysemys picta) Populations?
Source: PLoS One. 2014 May 23;9(5):e98414. doi: 10.1371/journal.pone.0098414 (PMC4032323; doi:10.1371/journal.pone.0098414)
Supplement: Table S4 — Results of t-tests comparing local and landscape variables at Road and No Road sites. Variables (a)–(d) weremeasured within a 5-m radius of the pond edge, variables (e)–(j) were measured at the surface of each pond, and variables (k)–(m) were measured within a 300-m radius of each pond. (DOC) [file pone.0098414.s005.doc]

**Table S4. Results of t-tests comparing local and landscape variables at Road and No Road**

**sites.** Variables (a) – (d) were measured within a 5-m radius of the pond edge, variables

(e) – (j) were measured at the surface of each pond, and variables (k) – (m) were measured

within a 300-m radius of each pond.

|  | **Variable** | **Site Type** | **Mean** | **SE** | **t** | **df** | **p** |
| --- | --- | --- | --- | --- | --- | --- | --- |
| (a) | % Forest cover | Road | 36 | 10.21 | 1.27 | 18 | 0.22 |
|  |  | No Road | 53.7 | 9.36 |  |  |  |
| (b) | % Grass cover | Road | 33.29 | 10.53 | -0.97 | 18 | 0.35 |
|  |  | No Road | 35.8 | 10.97 |  |  |  |
| (c) | % Shrub cover | Road | 12.5 | 7.76 | -0.22 | 18 | 0.83 |
|  |  | No Road | 10.5 | 4.86 |  |  |  |
| (d) | % Open ground | Road | 1 | 0.67 | -1.5 | 9 | 0.17 |
|  |  | No Road | 0 | 0 |  |  |  |
| (e) | % Open water | Road | 81.3 | 4.26 | 1.356 | 18 | 0.19 |
|  |  | No Road | 87.9 | 2.36 |  |  |  |
| (f) | % Emergent vegetation | Road | 7.5 | 1.71 | 0.075 | 18 | 0.94 |
|  |  | No Road | 7.7 | 2.07 |  |  |  |
| (g) | % Submerged aquatic vegetation | Road | 11.2 | 3.73 | -1.69 | 12 | 0.12 |
|  |  | No Road | 4.4 | 1.54 |  |  |  |
| (h) | Mean pH | Road | 9.46 | 0.24 | -1.6 | 18 | 0.13 |
|  |  | No Road | 8.96 | 0.19 |  |  |  |
| (i) | Mean temperature | Road | 25.51 | 0.38 | 0.582 | 18 | 0.57 |
|  |  | No Road | 25.88 | 0.5 |  |  |  |
| (j) | Mean conductivity | Road | 656.83 | 214.15 | -1.6 | 18 | 0.13 |
|  |  | No Road | 290.5 | 81.13 |  |  |  |
| (k) | % Forest cover | Road | 29.5 | 5.66 | 1.212 | 18 | 0.24 |
|  |  | No Road | 41.04 | 7.66 |  |  |  |
| (l) | % Crop cover | Road | 17.09 | 4.33 | 1.068 | 18 | 0.3 |
|  |  | No Road | 25.91 | 7.04 |  |  |  |
| (m) | % Urban area | Road | 7.45 | 6.55 | -0.92 | 18 | 0.37 |
|  |  | No Road | 1.36 | 1.14 |  |  |  |
| (n) | Distance to nearest body of water (m) | Road | 362 | 27.43 | -0.36 | 18 | 0.72 |
|  |  | No Road | 382 | 48.16 |  |  |  |
