# Supplementary material for: Do Roads Reduce Painted Turtle (Chrysemys picta) Populations?
Source: PLoS One. 2014 May 23;9(5):e98414. doi: 10.1371/journal.pone.0098414 (PMC4032323; doi:10.1371/journal.pone.0098414)
Supplement: Table S5 — Model summaries of simple linear regressions of the relationship between turtle relative abundance (log[Turtle Detections +1]) and predictor variables. Predictor variables (a)–(d) were measured within a 5-m radius of the pond edge, variables (e)–(j) were measured at the surface of each pond, and variables (k)–(m) were measured within a 300-m radius of each pond. (DOC) [file pone.0098414.s006.doc]

**Table S5. Model summaries of simple linear regressions of the relationship between turtle relative abundance [log(Turtle Detections)] and predictor variables.** Predictor variables (a) – (d) were measured within a 5-m radius of the pond edge, variables (e) – (j) were measured at the surface of each pond, and variables (k) – (m) were measured within a 300-m radius of each pond.

|  | **Habitat variable** | **R^2^** | **β** | **F** | **df** | **p** |
| --- | --- | --- | --- | --- | --- | --- |
| (a) | % Forest cover | 0.001 | 0.031 | 0.017 | 1, 18 | 0.897 |
| (b) | % Grass cover | <0.001 | -0.019 | 0.006 | 1, 18 | 0.938 |
| (c) | % Shrub cover | 0.006 | -0.077 | 0.108 | 1, 18 | 0.746 |
| (d) | % Open ground cover | 0.002 | -0.044 | 0.035 | 1, 18 | 0.854 |
| (e) | % Open water | 0.003 | -0.053 | 0.050 | 1, 18 | 0.825 |
| (f) | % Emergent vegetation | 0.113 | 0.336 | 2.290 | 1, 18 | 0.148 |
| (g) | % Submerged aquatic vegetation | 0.073 | -0.270 | 1.415 | 1, 18 | 0.250 |
| (h) | Mean pH | 0.005 | -0.067 | 0.082 | 1, 18 | 0.788 |
| (i) | Mean temperature | 0.052 | 0.229 | 0.996 | 1, 18 | 0.331 |
| (j) | Mean conductivity | 0.167 | -0.409 | 3.612 | 1, 18 | 0.073 |
| (k) | % Forest cover | 0.135 | 0.367 | 2.798 | 1, 18 | 0.112 |
| (l) | % Crop cover | 0.185 | -0.430 | 4.081 | 1, 18 | 0.060 |
| (m) | % Urban area | 0.061 | -0.246 | 1.161 | 1, 18 | 0.296 |
| (n) | Distance to nearest body of water (m) | 0.018 | 0.134 | 0.330 | 1, 18 | 0.573 |
